# Exploring the Effect of Phage Therapy in Preventing *Vibrio anguillarum* Infections in Cod and Turbot Larvae

**DOI:** 10.3390/antibiotics7020042

**Published:** 2018-05-16

**Authors:** Nanna Rørbo, Anita Rønneseth, Panos G. Kalatzis, Bastian Barker Rasmussen, Kirsten Engell-Sørensen, Hans Petter Kleppen, Heidrun Inger Wergeland, Lone Gram, Mathias Middelboe

**Affiliations:** 1 Marine Biological Section, University of Copenhagen, 3000 Helsingør, Denmark; nanna_ir@hotmail.com (N.R.); panos.kalatzis@bio.ku.dk (P.G.K.); 2 Department of Biology, University of Bergen, 5020 Bergen, Norway; anita.ronneseth@uib.no (A.R.); heidrun.wergeland@uib.no (H.I.W.); 3 Institute of Marine Biology, Biotechnology and Aquaculture, Hellenic Centre for Marine Research, 71003 Heraklion, Greece; 4 Department of Biotechnology and Biomedicine, Technical University of Denmark, 2800 Kongens Lyngby, Denmark; bbara@bio.dtu.dk (B.B.R.); gram@bio.dtu.dk (L.G.); 5 Fishlab, 8270 Højbjerg, Denmark; kes@fishlab.dk; 6 ACD Pharmaceuticals AS, 8376 Leknes, Norway; hans.kleppen@acdpharma.com

**Keywords:** *Vibrio anguillarum*, phage therapy, aquaculture, fish larvae, challenge trials

## Abstract

The aquaculture industry is suffering from losses associated with bacterial infections by opportunistic pathogens. *Vibrio anguillarum* is one of the most important pathogens, causing vibriosis in fish and shellfish cultures leading to high mortalities and economic losses. Bacterial resistance to antibiotics and inefficient vaccination at the larval stage of fish emphasizes the need for novel approaches, and phage therapy for controlling *Vibrio* pathogens has gained interest in the past few years. In this study, we examined the potential of the broad-host-range phage KVP40 to control four different *V. anguillarum* strains in Atlantic cod (*Gadus morhua* L.) and turbot (*Scophthalmus maximus* L.) larvae. We examined larval mortality and abundance of bacteria and phages. Phage KVP40 was able to reduce and/or delay the mortality of the cod and turbot larvae challenged with *V. anguillarum*. However, growth of other pathogenic bacteria naturally occurring on the fish eggs prior to our experiment caused mortality of the larvae in the unchallenged control groups. Interestingly, the broad-spectrum phage KVP40 was able to reduce mortality in these groups, compared to the nonchallenge control groups not treated with phage KVP40, demonstrating that the phage could also reduce mortality imposed by the background population of pathogens. Overall, phage-mediated reduction in mortality of cod and turbot larvae in experimental challenge assays with *V. anguillarum* pathogens suggested that application of broad-host-range phages can reduce *Vibrio*-induced mortality in turbot and cod larvae, emphasizing that phage therapy is a promising alternative to traditional treatment of vibriosis in marine aquaculture.

## 1. Introduction

*Vibrionaceae* is a genetic and metabolic diverse family of heterotrophic bacteria which are widespread in aquatic environments around the world [1]. Several vibrios are able to infect a wide range of aquatic animals and constitute therefore a large problem in aquaculture [2]. One of the most important is *Vibrio anguillarum*, which causes the disease vibriosis and is responsible for large-scale losses in the aquaculture industry [3,4]. Chemotherapy against vibriosis is associated with a major concern due to the risk of antibiotic-resistance developing in the pathogenic bacteria [5]. Vaccines against vibrio have been successful in preventing disease [6,7], however, they are often not useful at the larval stage, as the immune system is not fully developed. Therefore, alternative methods for the control and treatment of *V. anguillarum* infections in fish larvae and fry are needed. The use of bacteriophages (phages) has been explored in several studies as a treatment of pathogens in aquaculture [4,8,9,10,11,12,13]. Pereira et al. [4] and Mateus et al. [11] did in vitro assays with phages infecting different bacteria responsible for the diseases vibriosis and furunculosis and showed that both single-phage suspensions and phage cocktails could inactivate the bacteria [4,11]. However, often regrowth of phage tolerant bacteria was observed within 24 h after phage treatment [11,13]. Phage addition to shrimp larvae infected with *V. harveyi* caused a reduction in the pathogen load and significantly increased shrimp survival compared to untreated controls groups as well as parallel treatments with antibiotics [8,9]. Another study on zebrafish larvae infected with *V. anguillarum* [12] also found significantly enhanced larvae survival after phage addition. Successful phage treatment in Atlantic salmon (*Salmo salar* L.) infected with *V. anguillarum* strain PF4 was found for phage CHOED, resulting in complete elimination of pathogen-induced mortality when phages were added at a high multiplicity of infection [10]. Together, the previous experimental approaches demonstrate that phage therapy can be a feasible alternative method to control specific *Vibrio* pathogens in aquaculture. However, the use of phages is complicated by the fact that multiple strains of the *Vibrio* pathogens with different phage susceptibility patterns may coexist in aquaculture environments [14]. The implications of strain diversity for the efficiency of phage control may be overcome either by combining several phages which target a broad range of pathogenic hosts, or to use a broad-host-range phage which can infect multiple strains within a given species or even multiple species [15]. The phage KVP40 represents a broad-host-range phage which infects at least eight species of *Vibrio* sp. (*V. parahaemolyticus*, *V. alginolyticus*, *V. natriegens*, *V. cholerae*, *V. mimicus*, *V. anguillarum*, *V. splendidus*, and *V. fluvialis*) and one *Photobacterium* sp. (*P. leignathi*) [16]. All of these species contain a 26-kDa outer membrane protein named OmpK, which is a receptor for phage KVP40 [17].

The application of phages for controlling pathogens may be hampered by the development of phage resistance in the bacteria [18], and several mechanisms have been described in *V. anguillarum* which can eliminate or reduce bacterial sensitivity to phages and thus limit the efficiency and duration of phage control [19].

The aim of this study was to examine the effect of phage KVP40 on the survival of turbot and cod larvae challenged with four different *V. anguillarum* strains. Larval mortality and abundance of bacteria and phages were quantified to determine the potential of using phage KVP40 to control *V. anguillarum* infections during the early larval stage. In general, phage KVP40 was able to reduce or delay the mortality of both turbot and cod larvae in all the challenge trials and reduce larval mortality imposed by the background population of pathogens.

The results demonstrated that phage KVP40 reduced the mortality imposed by the added pathogens as well as other *Vibrio* pathogens already present in the environment during the initial 1–4 days of the experiment, emphasizing the potential of using phages to reduce turbot and cod mortality at the larval stage.

## 2. Results

### 2.1. Phage Effect on Turbot Mortality in Vibrio Challenge Trials

#### 2.1.1. Turbot Challenge Trial 1

**Figure 1:**
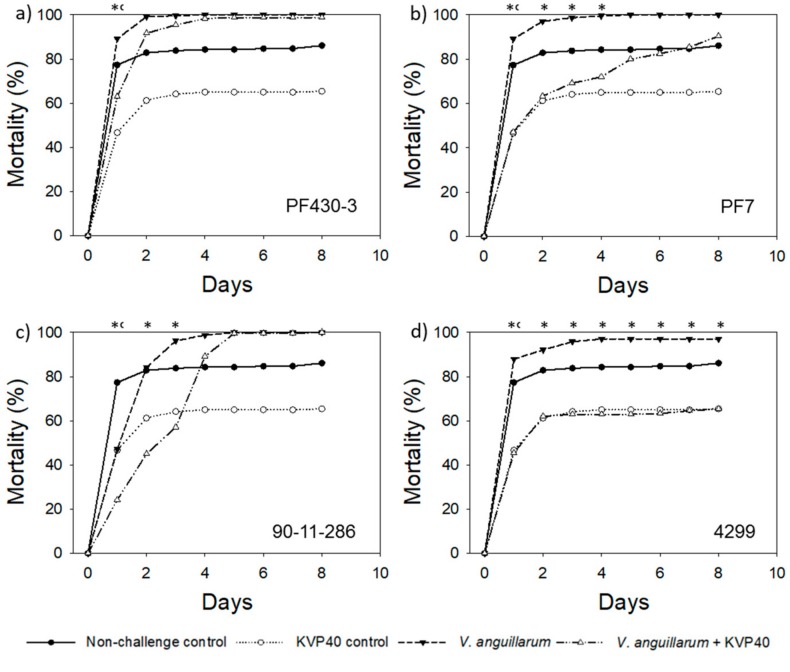
Cumulative percent mortality over time in turbot challenge trial 1: (**a**) strain PF430-3; (**b**) strain PF7; (**c**) strain 90-11-286; (**d**) strain 4299. Significant difference in mortality between cultures “*V. anguillarum*” and “*V. anguillarum* + KVP40” for individual time points is indicated by *. Significant difference in mortality between cultures “Nonchallenge control” and “KVP40 control” is indicated by ^c^.

In general, larval mortality was high in all treatments, including the nonchallenged controls where a maximum mortality of 86% (i.e., 103 dead larvae out of 120) was found (Figure 1), indicating that the eggs were associated with unknown bacterial pathogens prior to the challenge trial. Challenging the turbot eggs with *V. anguillarum* resulted in higher mortalities for all four strains (Figure 1), emphasizing that the added *V. anguillarum* pathogens increased larval mortality. Strain PF430-3 was the most virulent of the four strains, with 100% larval mortality after 3 days, whereas strains PF7, 90-11-286, and 4299 caused 97%–100% mortality after 4 and 5 days of challenge. Subsequent quantification of the abundance of colony forming bacteria in the water used for transportation of the fish eggs confirmed the presence of a microbial community associated with the eggs (see Section 2.5).

Despite the presence of other pathogen communities associated with the eggs/larvae, addition of phage KVP40 had a significant positive effect on larval survival in all the challenge treatments during all or part of the trials. When challenged with strain PF430-3, the maximum relative reduction in mortality was 29% (*p* < 0.05) one day after phage addition (Figure 1a; Table 1). The delay in mortality only lasted for 3 days, and the mortality reached almost 100% mortality at day 5 (Figure 1a). When challenged with strain PF7 or strain 90-11-286 (Figure 1b,c), the maximum phage-induced reduction in mortality was 47% obtained 1 and 2 days (Table 1), respectively, after addition of KVP40 and a significant effect of the phage on mortality was observed for 3–4 days (*p* < 0.05). The effect of phage addition was largest in the treatment group with strain 4299, where the larval mortality remained below 66% throughout the 8-day trial, corresponding to an average of 36% reduction in larval mortality compared to larvae challenged with *V. anguillarum* (*p* < 0.05) (Figure 1d).

Interestingly, larval mortality in the KVP40 controls (addition of phage but not *V. anguillarum*) showed the lowest larval mortality, reaching 65% at day 4 and remaining at that level (Figure 1). This significant reduction in mortality compared to the nonchallenged control (i.e., 86% mortality in larvae not exposed to *V. anguillarum* or phage) suggested that phage KVP40 was able to control part of the unknown pathogen community, thereby increasing the larval survival. This was later confirmed by analysis of phage susceptibility of bacteria initially associated with the eggs (see Section 2.5 below).

#### 2.1.2. Turbot Challenge Trial 2

The relatively high fraction of low-quality eggs and high mortality in the control group led us to repeat the challenge experiments in an attempt to optimize the egg quality and in order to verify the indications of positive effects of phages for larval mortality in replicate experiments.

**Figure 2:**
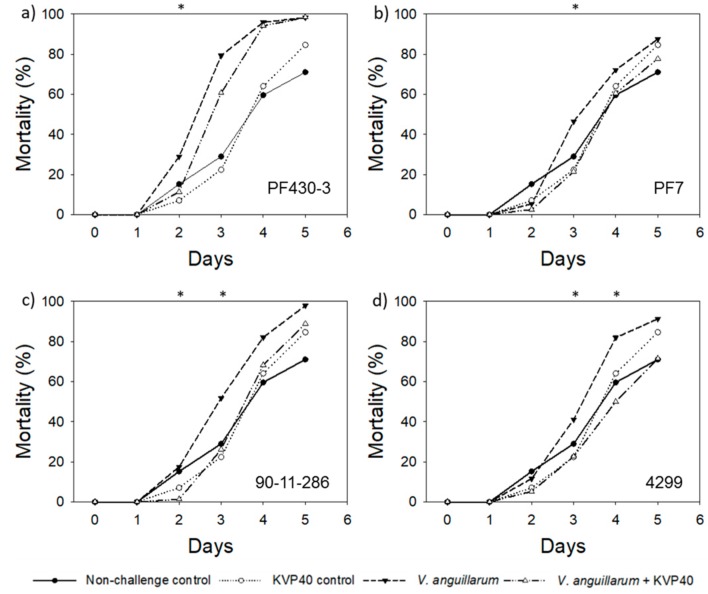
Cumulative percent mortality over time in turbot challenge trial 2: (**a**) strain PF430-3; (**b**) strain PF7; (**c**) strain 90-11-286; (**d**) strain 4299. Significant difference in mortality between cultures “*V. anguillarum*” and “*V. anguillarum* + KVP40” for individual time points is indicated by *.

Also in the second challenge trial with turbot larvae, a high mortality (71%) was observed in the nonchallenged control groups after 5 days (Figure 2), indicating pathogenic effects of the bacterial background community in the turbot eggs. In contrast to turbot challenge trial 1, the mortality caused by the background bacteria was not observed immediately, and mortality in the control groups gradually increased during the first 4 days, indicating growth of the pathogenic bacteria. Addition of *V. anguillarum* strains increased larval mortality in all four treatments, resulting in mortalities between 72% and 98% after 4–5 days of incubation. As in turbot challenge trial 1, addition of phage KVP40 had significant positive effects on the larval survival. However, in this case, the phage addition delayed the mortality by 2–4 days relative to the treatment with *V. anguillarum* alone.

When challenged with strain PF430-3, the addition of phages reduced mortality from 29% to 11% 2 days after phage addition (Figure 2a), corresponding to a maximum phage-mediated reduction in mortality of 60% (*p* < 0.05, Table 1). The delay in mortality lasted until day 4, where mortality approached 100% mortality as in the treatment without phage (Figure 2a). Phage addition to the larvae challenged with strain PF7 and strain 90-11-286 resulted in a significant 3-day delay in mortality with a maximum reduction in mortality of 53% and 92%, respectively, after 2–3 days relative to the larvae challenged with *V. anguillarum* alone (*p* < 0.05 (Figure 2b,c; Table 1). As in the turbot challenge trial 1, the larvae challenged with strain 4299 were best protected by phage addition, with a maximum relative reduction in mortality of 45% (*p* < 0.05) obtained 3 days after phage addition (Table 1), and a continued reduction in larval mortality of 22% relative to the larvae challenged with bacteria alone throughout the experiment (Figure 2d).

### 2.2. Abundance of Bacteria and Phages in Turbot Challenge Trial 2

**Figure 3:**
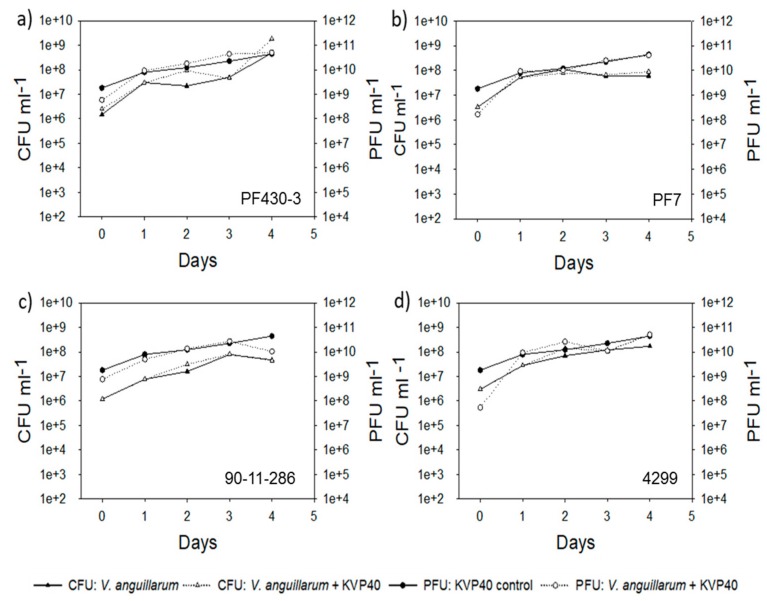
Bacterial abundance (CFU mL^−1^) and phage abundance (PFU mL^−1^) in turbot challenge trial 2: (**a**) strain PF430-3; (**b**) strain PF7; (**c**) strain 90-11-286; (**d**) strain 4299.

In all the treatments in turbot challenge trial 2, the total count of colony forming bacteria (CFU) increased exponentially over time for the first 2–4 days (Figure 3).

The number of infective KVP40 phages increased about 100-fold reaching 1–5 × 10^10^ PFU mL^−1^ in all the treatment groups where KVP40 was added, with no significant differences between cultures with and without the addition of *Vibrio* pathogens. This indicated that the background bacteria supported phage proliferation and that addition of *V. anguillarum* only had a minor effect on phage production.

### 2.3. Phage Effect on Cod Mortality in Vibrio Challenge Trials

#### 2.3.1. Cod Challenge Trial 1

**Figure 4:**
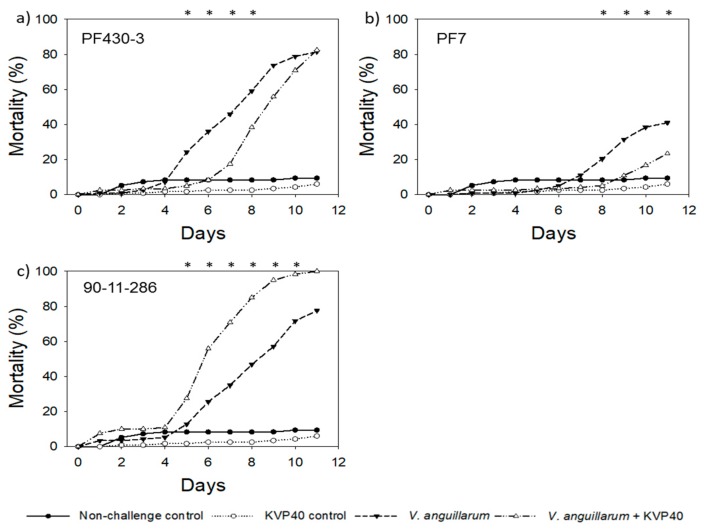
Cumulative percent mortality over time in cod challenge trial 1: (**a**) strain PF430-3; (**b**) strain PF7; (**c**) strain 90-11-286. Significant difference in mortality between cultures “*V. anguillarum*” and “*V. anguillarum* + KVP40” for individual time points is indicated by *.

The cod larvae mortality in the nonchallenged controls remained low throughout the trial (<10%) (Figure 4), and the addition of *Vibrio anguillarum* strains increased mortality significantly (Figure 4). Strain PF430-3 and strain 90-11-286 increased mortality to 82% and 78%, respectively, after 11 days (Figure 4a,c), whereas the mortality was 41% in the treatment with strain PF7 (Figure 4b).

The addition of phage KVP40 had significant positive effects on larval survival in the larvae exposed to strain PF430-3 and strain PF7. For strain PF430-3, the mortality was reduced from 24% to 5% in the phage added cultures after 5 days, corresponding to maximal relative reduction in mortality by phage KVP40 of 79% compared to the larvae only challenged with *V. anguillarum* (*p* < 0.05; Table 1). The significant phage-induced reduction in mortality lasted to day 8 (Figure 4a). Phage KVP40 addition to strain PF7 reduced relative larval mortality by 75% compared to the larvae only challenged with *V. anguillarum* (*p* < 0.05) after 8 days (Table 1), and the significant phage-mediated reduction in mortality remained throughout the 11-day trial (Figure 4b). Surprisingly, the addition of phage KVP40 increased larval mortality significantly in the cultures challenged with strain 90-11-286 with a maximum increase in mortality of 119 (*p* < 0.05) reached at day 6 (Figure 4c; Table 1). The negative effect of phage addition was significant from day 5 to day 10, with the mortality reaching 100% in the phage treated cultures at day 11.

Despite the low mortality in the nonchallenged control treatment, the reduced larval mortality in the phage KVP40 controls (addition of phage but not *V. anguillarum*) (<7%) compared with the nonchallenged control group without phages again indicated a positive effect of the phages in reducing the original pathogenic bacterial load in the trials.

#### 2.3.2. Cod Challenge Trial 2

**Figure 5:**
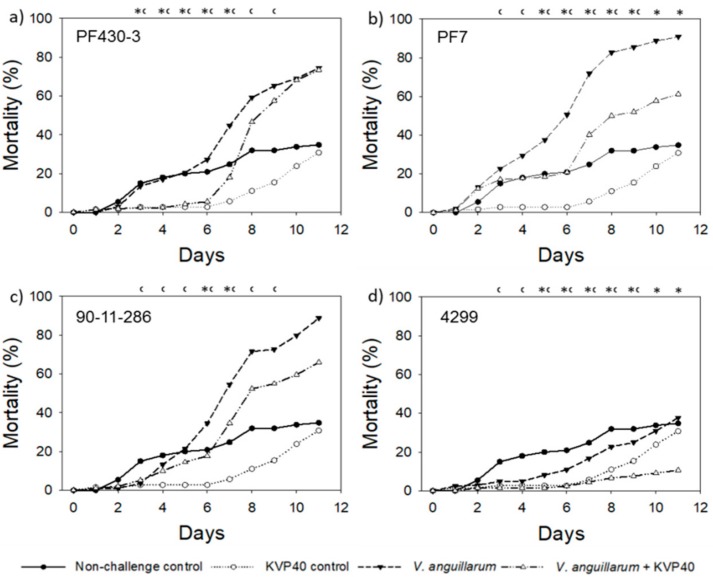
Cumulative percent mortality over time in cod challenge trial 2: (**a**) strain PF430-3; (**b**) strain PF7; (**c**) strain 90-11-286; (**d**) strain 4299. Significant difference in mortality between cultures “*V. anguillarum*” and “*V. anguillarum* + KVP40” for individual time points is indicated by *. Significant difference in mortality between cultures “Nonchallenge control” and “KVP40 control” is indicated by ^c^.

As for the turbot experiments, the challenge trials with cod were repeated to examine the reproducibility of the first results using a new batch of eggs. The second challenge trial with cod larvae confirmed the high virulence of strains PF430-3 and 90-11-286 obtained in cod challenge trial 1, whereas strain PF7 caused less mortality in cod challenge trial 2. Strain 4299 was not very virulent to the cod larvae (Figure 5). A gradual increase in mortality was observed in larvae challenged with strains PF430-3, PF7, and 90-11-286, which reached mortalities of 74% to 91% after 11 days post challenge (Figure 5a–c). Challenge with strain 4299 did not increase mortality compared to the nonchallenged control level, suggesting that this strain had very low virulence to cod (Figure 5d). The nonchallenged control showed an increase in mortality from 5% to 15% between days 2 and 3, followed by a more gradual increase to 35% mortality at day 11 (Figure 5).

Addition of phage KVP40 had a significant positive effect on cod larvae survival in all the treatments (Table 1). In the larvae challenged with strain PF430-3, phage addition kept larval mortality below 27% for 6 days, with a maximum reduction in mortality of 86% (*p* < 0.05) obtained 4 days after phage addition (Table 1). The reduced mortality lasted from day 2 to day 9, and after day 10 the mortality reached almost the same level as in the cultures without phages (Figure 5a). When challenged with strain PF7, the maximal effect of phage addition was a reduction in mortality of 59% (*p* < 0.05) obtained 6 days after phage addition (Table 1). The delay in mortality lasted throughout the trial, with the difference being significant from day 5 and onwards (Figure 5b). In the treatments challenged with strain 90-11-286, the maximal reduction in mortality was 49% (*p* < 0.05) obtained 6 days after phage addition (Table 1). The mortality then increased but remained below the nonphage treated group throughout the experiment (Figure 5c). Phage KVP40 very efficiently reduced mortality of larvae challenged with strain 4299, with a maximum reduction of 82% (*p* < 0.05) after 5 days (Table 1), and a significant reduction in mortality (mortality always < 12%) throughout the trial (Figure 5d).

The relatively high initial mortality in the nonchallenged control from day 1 to day 3 compared with corresponding nonchallenge control group in cod challenge trial 1, and compared with the lower and more gradual increase in mortality in the group challenged with strain 4299, suggested the presence of a high fraction of low-quality eggs in this specific control group. As in the previous trials, the phage-added controls showed a lower mortality than in the nonchallenged controls, again suggesting a positive effect of phage KVP40 in controlling other pathogens growing up during the trials (Figure 5).

### 2.4. Abundance of Bacteria and Phages in Cod Challenge Trials

#### 2.4.1. Cod Challenge Trial 1

**Figure 6:**
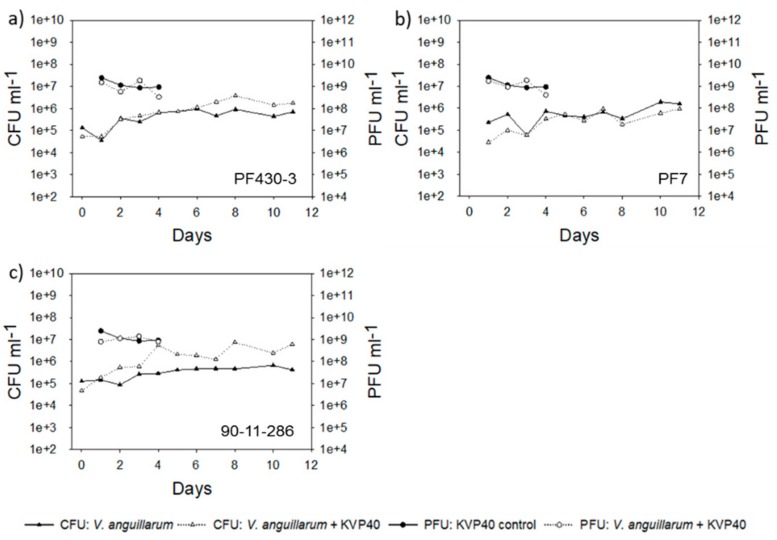
Bacterial abundance (CFU mL^−1^) and phage abundance (PFU mL^−1^) in cod challenge trial 1: (**a**) strain PF430-3; (**b**) strain PF7; (**c**) strain 90-11-286.

The total abundance of colony forming microorganisms increased approximately 10-fold in all *Vibrio* challenged larval groups from approx. 10^5^ to 10^6^ CFU mL^−1^ (Figure 6). Addition of phages only reduced the bacterial load in the strain PF7 challenged larval group and only during the first 2 days (Figure 6b). In contrast to this, total CFU counts increased after addition of phage KVP40 in larval groups challenged with strain PF430-3 and strain 90-11-286. Especially in the challenge with strain 90-11-286, a > 10-fold increase in colony forming bacteria was observed (Figure 6c) in accordance with the increased larval mortality in this treatment (Figure 4c). The phage abundance was approximately 10^7^ PFU mL^−1^ in all phage-added treatments and remained stable during the 4 days when PFU was measured.

#### 2.4.2. Cod Challenge Trial 2

**Figure 7:**
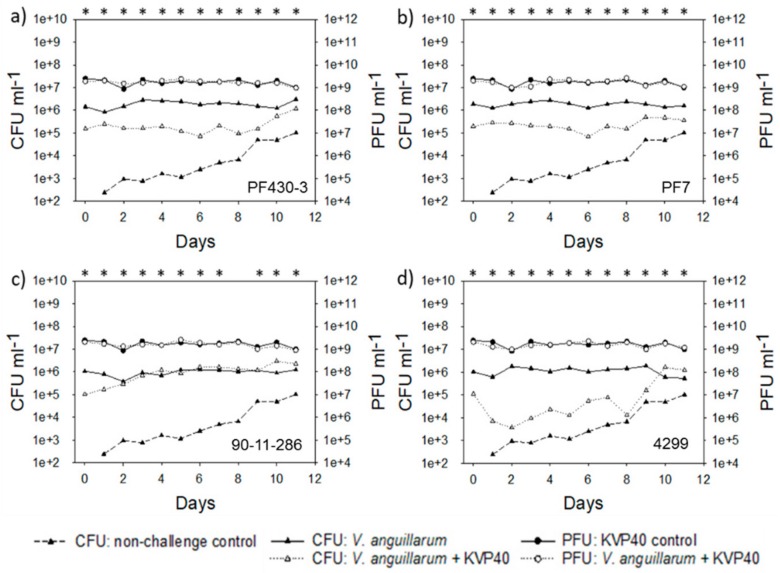
Bacterial abundance (CFU mL^−1^) and phage abundance (PFU mL^−1^) in cod challenge trial 2: (**a**) strain PF430-3; (**b**) strain PF7; (**c**) strain 90-11-286; (**d**) strain 4299. Significant difference in CFU between cultures “CFU: *V. anguillarum*” and “CFU: *V. anguillarum* + KVP40” for individual time points is indicated by *.

The *V. anguillarum* load was approximately 10-fold higher in the second than in the first cod challenge trial and the CFU counts were approximately 10^6^ CFU mL^−1^ in the *Vibrio* challenged groups (Figure 7).

In all the groups, addition of phage KVP40 reduced the bacterial counts significantly from day 0. In the groups challenged with strain PF430-3 and strain PF7, a significant phage-mediated reduction (approximately 1 log reduction) in the *V. anguillarum* pathogens was maintained for the first 8–9 days, followed by an increase in total CFU which then reached values close to the bacteria-alone group at day 11 (Figure 7a,b). For the group challenged with strain 90-11-286, phage reduction of the *Vibrio* pathogen was rather short. After 3 days, the bacterial abundance had reached the same level as in the bacteria-only group (Figure 7c). In the group challenged with strain 4299, the addition of phage KVP40 caused a 100-fold reduction in total CFU counts, indicating a strong phage control of the pathogen. However, after day 8, total bacterial cell counts increased 100-fold and reached numbers similar to the group without phage (Figure 7d). Phages were added at an initial concentration of 1.75 × 10^9^ PFU mL^−1^ and the abundance of phage remained stable throughout the trial, both in the absence and presence of the *Vibrio* hosts (Figure 7).

### 2.5. Abundance and Phage KVP40 Susceptibility of Bacterial Background Communities Associated with the Turbot Eggs

During the second turbot trial, the abundance of colony-forming bacteria in water used for transportation of the fish eggs was determined to shed light on the observed positive effect of phage KVP40 on unchallenged control groups. Different general and *Vibrio*-promoting growth media were used. In all the experiments, there was a high load of bacteria associated with the eggs, and a general increase in their abundance over time was found (Table 2). The high abundance of colonies growing on TCBS plates (up to >10^8^ CFU mL^−1^) indicated that a large fraction of these background communities were presumptive *Vibrio* or *Vibrio*-related species.

**Figure 8:**
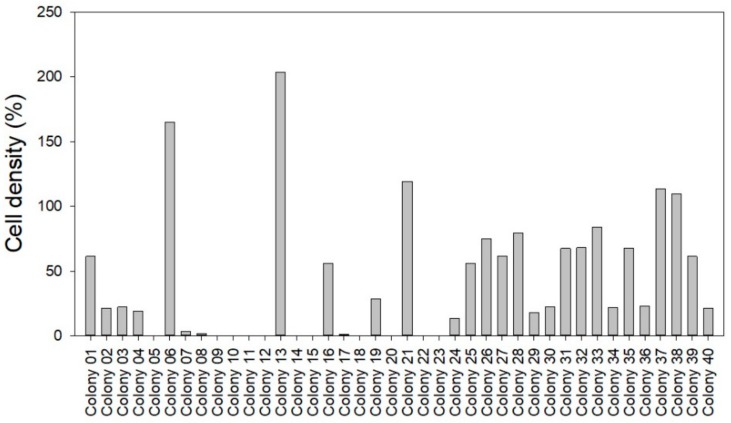
Quantification of phage KVP40-induced inhibition/promotion of cell growth in cultures of bacteria isolated from water used for transport of eggs used in turbot challenge trial 1. Phage-induced growth inhibition/promotion was determined as the percent cell density in cultures added phage KVP40 relative to control cultures without phage KVP40 (100%) after 3 h incubation.

The susceptibility to phage KVP40 was tested in 40 isolates obtained from the water containing the turbot eggs during transportation used for challenge trial 1 by quantification of the growth reduction relative to a control culture without phage KVP40 (Figure 8). The results showed that 35 out of 40 isolated showed a growth reduction, indicating that the majority of the colony-forming cells originating from the water used for transporting the eggs were susceptible to phage KVP40.

## 3. Discussion

In general, the addition of phage KVP40 reduced or delayed the mortality of turbot and cod larvae challenged with *V. anguillarum*, with the largest effect observed for strain 4299, where the relative turbot and cod mortality was reduced by 22–33% and 72%, respectively, by the end of the experiment. In most of the challenges, the positive effect of phage KVP40 addition on larval survival was maintained throughout the incubation period. However, incubation with strain PF430-3 showed a temporary effect of phage addition on mortality and larval mortality reached the same level as in the bacterial challenges (without phage) after 4–10 days. Since the phage was maintained in high concentrations throughout the experiment, it is likely that strain PF430-3 was protected against infection, which supports previous observations that strain PF430-3 can reduce its susceptibility to phage KVP40 by forming aggregates or biofilm, creating spatial refuges [20].

In addition to the specific *V. anguillarum* pathogens, other pathogens already associated with the fish eggs prior to the experiments were present in the experiments. This allowed an assessment of the effects of phages on both the mortality caused by the *V. anguillarum* strains and the mortality imposed by the natural background pathogen communities. The decrease in mortality recorded for all the phage controls (without *V. anguillarum*) compared to the nonchallenge controls (without phage and *V. anguillarum*) demonstrated a strong effect of phage KVP40 on the initial bacterial pathogen communities associated with the eggs. This was supported by the observation that >85% of the isolated colonies originating from the background bacterial community were susceptible to phage KVP40.

Despite the large fraction of phage susceptible strains, the bacterial abundance increased in all the incubations over time, and only in cod challenge trial 2 did addition of phage KVP40 reduce the bacterial abundance for multiple days. This suggested that during the experiment, pathogens that were not infected by KVP40 (i.e., non-*Vibrio* pathogens and possibly phage-resistant *V. anguillarum* strains) replaced the phage susceptible strains, and thus were the main cause of mortality in the experiments. This was supported by the increased effects of phages on mortality in cod challenge trial 2, where the eggs were pretreated with 25% glutaraldehyde. These results emphasized that the growth of other pathogens than *V. anguillarum* was the main cause of mortality in the experiments that were not pretreated with glutaraldehyde, and that phage KVP40 was able to significantly reduce mortality imposed by the added *V. anguillarum* strains.

Consequently, even though the presence of a bacterial background pathogen community masked the effect of phage KVP40 on the added *V. anguillarum* strains, it at the same time provided a more realistic demonstration of how the addition of phage KVP40 will affect an infected aquaculture system. These results emphasized the potential of phage KVP40 to control not only the added host strains but also a broader range of pathogens present in the rearing facilities. Similar results were obtained for the two broad-host-range KVP40-like phages *φ*St2 and *φ*Grn1 infecting the fish pathogens *V. alginolyticus* [21]. These phages were able to reduce the natural *Vibrio* load present in *Artemia* live feed cultures used in fish hatcheries. The current study is, however, the first demonstration of a positive effect of phage application on larval survival by reducing the natural microbiota, rather than exclusively focusing on the effects of one added pathogen. While the composition of the background microbiota was not analyzed in the current study, previous studies have found that bacterial communities associated with cod and turbot eggs in rearing units were dominated by *Pseudomonas*, *Alteromonas*, *Aeromonas*, and *Flavobacterium* [22], but also *Vibrio* has been shown to be prevalent in these environments [23]. In our study, the high fraction of bacteria growing on *Vibrio*-selective TCBS medium combined with the high susceptibility to phage KVP40 suggested that the background bacterial community was dominated by *Vibrio* or *Vibrio*-related species, as the phage KVP40 has been shown to infect at least eight *Vibrio* species and one *Photobacterium* [16]. This was also supported by preliminary analysis of the microbiome associated with the turbot eggs used in challenge trial 2, which showed dominance of *Vibrio* species (Dittmann, unpublished results). The differences in mortality in the control treatments (nonchallenged control and KVP40 control) between different experiments may therefore reflect differences in the composition of background bacterial community, representing differences in virulence and KVP40 susceptibility. Further, higher incubation temperature of the turbot than cod eggs may also have increased bacteria-induced mortality in the turbot experiments. In one of the treatments (cod challenge trial 1 with strain 90-11-286), addition of phage KVP40 increased larval mortality (Figure 4c). Specific secondary metabolites or toxins released during cell lysis may potentially inhibit larval growth [24]. However, since this was not observed in any of the other treatments, it is not likely that the viral lysates affected the cod larvae. Alternatively, the viral lysates may have stimulated growth of other specific pathogens already present in the experiment, as also indicated by the enhanced bacterial growth in the phage added culture (Figure 6c). Previous studies have shown that lysogenization of *V. harveyi* with phage VHS1 increased the virulence of the bacterium against black tiger shrimp (*Penaus monodon*) by the phage encoded toxin associated with hemocyte agglutination ([25]). There has not been any indication of lysogenization of *Vibrio* pathogens with phage KVP40, and the production of a KVP40-encoded toxin is therefore not a likely explanation for the observed increase in larval mortality in this experiment.

Our results support previous attempts to control pathogens in aquaculture by use of phages. A challenge trial in Atlantic salmon using *V. anguillarum* strain PF4, a close relative to strain PF430-3 used in the current study [13], showed 100% survival using the phage CHOED, independent of the original multiplicity of infection (MOI) [10]. The efficiency of this phage on fish survival compared to the current study most likely relates to the fact that larger fish are more robust against infections by co-occurring pathogens than larvae. A delay in mortality after phage addition was also observed by Imbeault et al. [26] and Verner-Jeffreys et al. [27] in brook trout and Atlantic salmon, respectively, infected with *A. salmonicida* using different phages. While Imbeault et al. [26] were able to delay the onset of disease and reduce the mortality to 10%, Verner-Jeffreys et al. [27] also demonstrated a delay in the mortality, but only observed a temporary effect of the phages in survival.

Previous in vivo challenge studies with a positive outcome of phage therapy were conducted on >5 day old larvae [12] or fish averaging 15–25 grams [10], while our study was conducted on eggs which hatched during the course of the challenge trials. Eggs and newly hatched larvae are more sensitive to the infection by pathogenic *V. anguillarum* and other pathogens than late stages due to the inefficient protection provided by the intestinal microflora associated with their gut mucosa, which constitutes a primary barrier [28]. Despite the general frailty of newly hatched larvae, we demonstrated a significant phage-mediated reduction in mortality of cod and turbot larvae in experimental challenge trials with *V. anguillarum* pathogens in combination with the natural pathogenic bacteria associated with the incubated fish eggs. These results emphasize that phage therapy is a promising approach to reduce pathogen load and mortality in marine larviculture.

## 4. Materials and Methods

### 4.1. Bacterial Strains and Growth Conditions

The four *V. anguillarum* strains—PF4303-3, PF7, 90-11-286, and 4299—used in this study were isolated in Chile, Denmark, and Norway [10,13,29,30]. The bacteria were stored at −80 °C in Luria-Bertani (LB) medium with 15% glycerol. Before each assay, the strains were inoculated on LB plates and grown overnight at 24 °C. Then, one colony was transferred to 4 mL LB medium and grown overnight at 24 °C with agitation (200 rpm).

### 4.2. Phage Infectivity and Production

The broad-host-range phage KVP40 [16], which previously has been shown to infect the *V. anguillarum* strains PF430-3, 90-11-286, and 4299 [13], was tested on *V. anguillarum* strain PF7 using the double-layer agar assay [14] with minor modifications. The double-layer agar assay in brief: 100 µL phage lysate was mixed with 300 µL bacterial cells and incubated for 30 min at 24 °C. The mixture was added to 4 mL of 45 °C top agar (LB with 0.4% agar) and poured onto a LB 1.5% agar plate, which was placed for incubation at 24 °C overnight. The next day, the presence of phages in the form of clear plaques in the top agar was detected. KVP40 was produced and purified by ACD Pharmaceuticals AS (Leknes, Norway).

### 4.3. Eggs and Larvae

Eggs from turbot and cod were used in the challenge trials. The eggs for turbot challenge trial 1 were obtained from Stolt Sea Farm (Galicia, Spain), with 48 h of transport before conducting the challenge trial at the University of Bergen (Bergen, Norway). The eggs for turbot challenge trial 2 were obtained from France Turbot, hatchery L’Epine (Noirmoutier Island, France), with 24 h of transport before conducting the challenge trial at the Technical University of Denmark (Lyngby, Denmark). The eggs for cod challenge trial 1 and cod challenge trial 2 were obtained from the Institute of Marine Research, Austevoll Research Station (Storebø, Norway), with 1 hour of transport before conducting the challenge trial at the University of Bergen (Bergen, Norway). The eggs in cod challenge trial 2 were disinfected with 25% glutaraldehyde at the Institute of Marine Research, Austevoll Research Station before being transported to the University of Bergen for the challenge trial.

### 4.4. Phage Therapy Assays

Challenge trials with turbot and cod larvae were established as outlined in Table 3. For each of the *V. anguillarum* strains tested, eggs were distributed in 10 24-well dishes with 2 mL sterile filtered (0.2 µm) and autoclaved, oxygenated 80% sea water and 1 egg well^−1^. In group 1 (*V. anguillarum* only), five 24-well plates were inoculated with 100 µL *V. anguillarum* culture in each well. Prior to addition, the bacterial culture had been grown overnight, washed twice in sterile sea water (ssw), and resuspended in ssw to a final concentration of 0.5–1 × 10^6^ CFU mL^−1^. In group 2 (*V. anguillarum* + phage KVP40), five 24-well plates were inoculated with *V. anguillarum* as above and 50 µL of phage KVP40 was added to each well to a final concentration of 0.5–8 × 10^8^ PFU mL^−1^, resulting in a multiplicity of infection (MOI) of ~5–100. The five 24-well plates in group 3 (nonchallenged control) were only inoculated with 100 µL autoclaved, oxygenated 80% ssw, whereas in group 4 (phage KVP40 control), each well also contained 50 µL of phage KVP40. Plates were then incubated in an air-conditioned room of 15.5 °C and 5.5 °C for turbot and cod, respectively, which are optimal conditions for larval development in the two species. The eggs in groups 1, 2, and 4 had bacteria and/or phages added to them immediately after their distribution in the wells (=day 0 of the experiment). Due to large variation in the viability of the eggs used for the experiment, the challenge trials were done twice for both fish species in an attempt to confirm the results at different egg qualities. The challenge trials lasted for 8 days for turbot challenge trial 1, 5 days for turbot challenge trial 2, and for 11 days for cod. The mortality was monitored daily. The quality of the eggs varied considerably depending on transportation time and handling, resulting in differences in egg mortality prior to hatching. The initial egg mortality was calculated for each 24-well plate and then averaged for all 50 24-well plates used in the individual experiments. Of the 1200 eggs used in each experiment, the average fraction of eggs that died prior to hatching amounted to 0% and 30.3% in turbot challenge trials 1 and 2, respectively, and 4.9% and 23.2% in cod challenge trials 1 and 2, respectively. These eggs were excluded from the analysis. The effect of phage addition on larval mortality was calculated as a relative reduction [31], corresponding to the reduction in mortality in treatments to which both phage KVP40 and *V. anguillarum* were added relative to the mortality in treatments with *V. anguillarum* alone (i.e., the difference in mortality between the two treatments in percentage of the mortality in the incubations without phage.

The concentration of bacteria and phages was monitored daily except in turbot challenge trial 1, where neither was monitored. In turbot challenge trial 2, the concentrations were only monitored for half of the experiment, while the phage concentration was only monitored for 3 days in cod challenge trial 1. To determine the bacterial concentration, dilutions were inoculated on LB agar plates (in cod challenge trial 2, the dilutions were inoculated on marine agar plates and on selective thiosulfate-citrate-bile salts-sucrose (TCBS) plates), which incubated overnight at 24 °C. To determine the phage concentration, the double-layer agar assay was used as described earlier. The culture medium was LB, the host strain was *V. anguillarum* strain PF430-3 Δ*vanT* [19], and the plates were incubated overnight at 24 °C.

### 4.5. Bacterial Background Community and Susceptibility Assays

In order to characterize the bacterial background, different media were used in the challenge trials. The water used for the transport of the eggs in turbot challenge trial 1 was spread on TCBS plates at day 4. A total of 40 colonies were picked and transferred to LB medium and grown overnight at 24 °C with agitation (200 rpm). The bacteria had their optical density at 600 nm (OD_600_), measured using Novaspec Plus Visible Spectrophotometer after 1 hour in the presence and in the absence of KVP40. The sterile 80% sea water with the live nonchallenged control larvae in turbot challenge trial 2 were inoculated on LB, TCBS, and marine agar plates at day 11. The plates incubated overnight at 24 °C before determining the bacterial concentration. Throughout cod challenge trial 2, the bacterial concentration was determined on both marine agar and TCBS plates.

### 4.6. Statistical Analysis

Differences between challenged larvae with and without phage therapy and between the controls (nonchallenge control and KVP40 control) for each time point were analyzed by chi-squared tests using the software R (R foundation for statistical computing). A value of *p* < 0.05 were considered statistically significant.

## 5. Conclusions

The significant positive effect of phage KVP40 on larval survival during hatching and initial growth observed in the current experiment demonstrates the potential in using phages to reduce pathogen load in cod and turbot hatcheries and may also be a strategy to improve egg quality and survival during transport from egg producers to hatcheries. It is obvious, however, that the effect of the phage addition on mortality is temporary, and we suggest that a more efficient and long-term control of the pathogens may be obtained using a cocktail of different phages that target a broader range of pathogens.

## Figures and Tables

**Table 1:** Overview of the percent reduction in mortality caused by phage KVP40 addition in the four experiments. The maximum relative reduction and reduction at the end of the experiment (final) is shown.

	Relative Reduction * in Larval Mortality in the Presence of Phages (%)							
*V. anguillarum* Strains	Turbot Challenge Trial				Cod Challenge Trial			
	1		2		1		2	
	Max.	Final	Max.	Final	Max.	Final	Max.	Final
**PF430-3**	29	N/S ^1^	60	N/S ^1^	79	N/S ^1^	86	N/ ^1^
**PF7**	47	N/S ^1^	53	N/S ^1^	75	43	59	32
**90-11-286**	47	N/S ^1^	92	N/S ^1^	−119	N/S ^1^	49	N/S ^1^
**4299**	48	33	45	N/S ^1^	N/D ^2^	N/D ^2^	82	72

* The relative reduction in mortality is calculated as difference in mortality between *V. anguillarum* and *V. anguillarum* + phage treatment, divided by the mortality in the *V. anguillarum* treatment. ^1^ N/S: not significant, ^2^ N/D: not determined.

**Table 2:** Abundance of the bacterial background community (CFU mL^−1^) associated with the fish eggs, in turbot challenge trial 2, and cultured on different media. Day 0: water the eggs were transported in for 24 h; Day 11: water in the wells of the live nonchallenged larvae.

Growth Substrate	Day 0 (CFU mL^−1^)	Day 11 (CFU mL^−1^)
LB media	2 × 10^7^	9.39 × 10^6^
TCBS media	2 × 10^6^	1.5 × 10^8^
Marine agar	N/D ^1^	2.89 × 10^8^

^1^ N/D: not determined.

**Table 3:** Experimental design and addition *V. anguillarum* and phage KVP40.

Group	Treatment	*V. anguillarum* (CFU mL^−1^)	Phage KVP40 (PFU mL^−1^)	Replicate Wells
1	*V. anguillarum* only	0.5–1 × 10^6^	-	5 × 24 wells × 4 strains
2	*V. anguillarum* + phage KVP40	0.5–1 × 10^6^	0.5–12 × 10^8^	5 × 24 wells × 4 strains
3	Nonchallenge control	-	-	5 × 24 wells
4	Phage KVP40 control	-	0.5–12 × 10^8^	5 × 24 wells
